# Suppression of MyD88 disturbs gut microbiota and activates the NLR pathway and hence fails to ameliorate DSS-induced colitis

**DOI:** 10.1093/pcmedi/pbae013

**Published:** 2024-06-06

**Authors:** Jun-hua Li, Yu Chen, Zheng-hao Ye, Li-ping Chen, Jia-xin Xu, Jian Han, Lin Xie, Shuai Xing, De-an Tian, Ursula Seidler, Jia-zhi Liao, Fang Xiao

**Affiliations:** Department of Nephrology, Tongji Hospital, Tongji Medical College, Huazhong University of Science and Technology, Wuhan 430030, China; Department of Gastroenterology, Tongji Hospital, Tongji Medical College, Huazhong University of Science and Technology, Wuhan 430030, China; Department of Gastroenterology, Tongji Hospital, Tongji Medical College, Huazhong University of Science and Technology, Wuhan 430030, China; Department of Gastroenterology, Tongji Hospital, Tongji Medical College, Huazhong University of Science and Technology, Wuhan 430030, China; Department of Gastroenterology, Tongji Hospital, Tongji Medical College, Huazhong University of Science and Technology, Wuhan 430030, China; Department of Gastroenterology, Tongji Hospital, Tongji Medical College, Huazhong University of Science and Technology, Wuhan 430030, China; Institute of Organ Transplantation, Tongji Hospital, Tongji Medical College, Huazhong University of Science and Technology, Wuhan 430030, China; Department of Gastroenterology, Tongji Hospital, Tongji Medical College, Huazhong University of Science and Technology, Wuhan 430030, China; Department of Gastroenterology, Tongji Hospital, Tongji Medical College, Huazhong University of Science and Technology, Wuhan 430030, China; Department of Gastroenterology, Hepatology and Endocrinology, Hannover Medical School, Hannover 30625, Germany; Department of Gastroenterology, Tongji Hospital, Tongji Medical College, Huazhong University of Science and Technology, Wuhan 430030, China; Department of Gastroenterology, Tongji Hospital, Tongji Medical College, Huazhong University of Science and Technology, Wuhan 430030, China

**Keywords:** microbiota, innate immunity, myeloid differentiation factor 88, MyD88 inhibitor, NOD-like receptor, inflammatory bowel disease

## Abstract

**Background:**

Myeloid differentiation factor 88 (MyD88) is the core adaptor for Toll-like receptors defending against microbial invasion and initiating a downstream immune response during microbiota–host interaction. However, the role of MyD88 in the pathogenesis of inflammatory bowel disease is controversial. This study aims to investigate the impact of MyD88 on intestinal inflammation and the underlying mechanism.

**Methods:**

MyD88 knockout (MyD88^−/−^) mice and the MyD88 inhibitor (TJ-M2010-5) were used to investigate the impact of MyD88 on acute dextran sodium sulfate (DSS)-induced colitis. Disease activity index, colon length, histological score, and inflammatory cytokines were examined to evaluate the severity of colitis. RNA transcriptome analysis and 16S rDNA sequencing were used to detect the potential mechanism.

**Results:**

In an acute DSS-colitis model, the severity of colitis was not alleviated in MyD88^−/−^ mice and TJ-M2010-5-treated mice, despite significantly lower levels of NF-κB activation being exhibited compared to control mice. Meanwhile, 16S rDNA sequencing and RNA transcriptome analysis revealed a higher abundance of intestinal *Proteobacteria* and an up-regulation of the nucleotide oligomerization domain-like receptors (NLRs) signaling pathway in colitis mice following MyD88 suppression. Further blockade of the NLRs signaling pathway or elimination of gut microbiota with broad-spectrum antibiotics in DSS-induced colitis mice treated with TJ-M2010-5 ameliorated the disease severity, which was not improved solely by MyD88 inhibition. After treatment with broad-spectrum antibiotics, downregulation of the NLR signaling pathway was observed.

**Conclusion:**

Our study suggests that the suppression of MyD88 might be associated with unfavorable changes in the composition of gut microbiota, leading to NLR-mediated immune activation and intestinal inflammation.

## Introduction

Inflammatory bowel disease (IBD) is a chronic and relapsing inflammatory disorder predominantly affecting the gastrointestinal tract, including ulcerative colitis and Crohn's disease [1]. In recent years, the incidence of IBD has increased globally, which brings a rising burden on health care [2]. Although it is generally accepted that IBD is a result of microbial dysbiosis, exaggerated immune response, disrupted intestinal barrier, and genetic susceptibility, the precise etiology of IBD remains elusive [1, 3]. This is the main reason that current therapeutic strategies for IBD show limited efficacy but substantial adverse effects in achieving long-term remission [4]. Dysregulated interaction between intestinal microbiota and the immune system plays a pivotal role in the initiation and progression of intestinal inflammation [5].

Invading microbiota can stimulate immune responses through various approaches. Dendritic cells located in the lamina propria can directly interact with pathogens and serve as antigen-presenting cells to stimulate adaptive T cells [6]. Diverse metabolites from microbiota can regulate immune reaction through metabolite-specific receptors such as Takeda G protein-coupled receptor 5 (TGR5) and Aryl-hydrocarbon receptor (AhR) [7]. Imbalance between gut commensal and pathogenic bacteria may lead to immune activation responsible for intestinal inflammation. Pattern-recognition receptors (PRRs) are the major receptors for a host to recognize the invading microbiota through conserved molecular structures termed pathogen-associated molecular patterns (PAMPs). Toll-like receptors (TLRs) and nucleotide oligomerization domain (NOD)-like receptors (NLRs) are the two main families of PRRs [8]. TLRs are mainly localized in the cell membrane and are capable of recognizing a broad range of exogenous PAMPs, while NLRs specialize in defense against intracellular microbes [9]. The recognition of TLRs can initiate a cascade of innate and adaptive immune responses and play a central part in the pathogenesis of IBD [9, 10].

The molecule myeloid differentiation factor 88 (MyD88) is the core adaptor of TLRs and one of major mediators to activate the nuclear factor kappa-B (NF-κB) pathway [11]. Patients with IBD showed enhanced activation of MyD88 signaling in intestinal epithelial cells [12]. The upregulation of MyD88/NF-κB signal induces the production of multiple proinflammatory cytokines in IBD [13]. The differentiation and effector function of T cells also require MyD88. It was reported that deletion of MyD88 in CD4^+^ T cells resulted in defective differentiation of Th17 and decreased secretion of interleukin (IL)-17 in mice with colitis [14]. These innate and adaptive immune responses mediated by MyD88 promote the development of intestinal inflammation and aggravate epithelial injury in IBD patients [13]. Thus, MyD88 was regarded as a possible therapeutic target for IBD [11]. However, the role of MyD88 in the development of IBD is complex and controversial. In addition to regulating immune responses, MyD88 is also important in modulating intestinal bacterial clearance, epithelial restoration, mucosal permeability, and even microbial composition [15– 18]. Several animal experiments showed that deletion of MyD88 could not mitigate intestinal inflammation and even increased tissue susceptibility to colitis [19–21]. Therefore, further research is required to investigate the effects of MyD88 on intestinal inflammation and the underlying mechanism.

This study set out to analyze the *in vivo* impacts of MyD88 deficiency or inhibition by a MyD88 inhibitor on acute intestinal inflammation in terms of the crosstalk between gut microbiota and the innate immune system. Our results revealed a potential role of MyD88 in shaping the structure of gut microbiota and a cooperation mechanism of TLR- and NLR-mediated immunological pathways.

## Methods

### Mice

MyD88^−/−^ mice with C57BL/6J background and their wild-type (WT) littermates were obtained from Nanjing Medical University, Nanjing, China. C57BL/6J female mice (6–8 weeks old) were purchased from Beijing HFK Bioscience Co., Ltd. All mice were bred and maintained under conventional laboratory conditions at the animal center of Tongji Medical College. Experimental protocols were approved by the Institute Animal Care and Use Committee at the Tongji Hospital, Wuhan, China. WT C57BL/6J female mice (6–8 weeks old) were purchased from HFK Bioscience and were randomly divided into control and treatment groups. All mice were bred under conventional laboratory conditions at the animal center of Tongji Medical College. Experimental protocols were approved by the Institute Animal Care and Use Committee at the Tongji Hospital, Wuhan, China.

### Regents

The MyD88 inhibitor TJ-M2010-5 (TJ5) was generated in the School of Pharmacy, Tongji Medical College, Huazhong University of Science and Technology, Wuhan, China (WIPO Patent Application Number: PCT/CN2012/070 811) and kindly provided by Prof. Ping Zhou. NLRs inhibitor NOD-IN-1 (NI1) was purchased from MedChemExpress (MJ, USA). Imipenem (Merck Sharp & Dohme Corp. U.S.A) and vancomycin (VANCOCIN ITALIA S.R.L) were obtained from Tongji Hospital. Antibodies used for western blotting were purchased from Cell Signaling Technology (MA, USA), ABclonal technology (Wuhan, China) and Proteintech (Chicago, IL< USA).

### Induction of colitis and treatment

Acute colitis was induced in mice with C57BL/6J background by treating them with 3% (wt/vol) dextran sulphate sodium (DSS, 36–50 kDa; MP Biomedicals, USA) in their drinking water for 7 days. The MyD88 knockout mice and their littermates were organized into the following groups: WT group, WT-DSS group, MyD88^−/−^ group, and MyD88^−/−^-DSS group. For the MyD88 inhibition study, C57BL/6J mice were intraperitoneally injected with 50 mg/kg of TJ5 daily from one day before the first DSS administration to the seventh day of DSS challenge. H_2_O or PBS was used as a solvent for DSS and the MyD88 inhibitor TJ5, serving as the control for DSS or TJ5, respectively. To eliminate the gut microflora, mice were given imipenem (1 g/l) and vancomycin (1 g/l) in drinking water 3 days before and during DSS administration. The NI1 was administrated at a dose of 20 mg/kg per day via intraperitoneal injection.

### Disease activity index and histological score

Mice were monitored daily for weight, stool consistency, and rectal bleeding. The disease activity index (DAI) was calculated under the guidance of a previously established scoring system (supplementary Table S1, see online supplementary material) [22].

The colonic tissues were fixed in 4% formaldehyde, paraffin-embedded, then sectioned at 3–6 μm and stained with hematoxylin and eosin (H&E). Each section was evaluated by three blinded researchers through the histological score (HS) system (supplementary Table S2, see online supplementary material) [23].

### RNA extraction and RT-PCR

Total RNA was extracted by lysing colonic tissue with Trizol reagent. Complementary DNA (cDNA) was synthesized from 0.5 µg of total RNA using a reverse transcriptase kit (Vazyme, Nanjing, China), and RT-PCR was performed using SYBR Green qPCR Master Mix (Vazyme) on an ABI StepOne Real-Time PCR system (Thermo Fisher Scientific, MA, USA). All primers were synthesized by Tsingke Biological Technology (Beijing, China) (supplementary Table S3, see online supplementary material). The relative mRNA expression levels of target genes were analyzed by using the 2^−ΔΔCT^ method with β-actin as the reference gene.

### Western blotting

Colonic protein was extracted using a protein extraction kit (Solarbio, Beijing, China). The protein lysates were run on SDS-PAGE and transferred to a polyvinylidene fluoride (PVDF) membrane (Millipore, Darmstadt, Germany). The PVDF membranes were blocked with 5% non-fat milk and were incubated at 4°C overnight. Afterwards, the membranes were incubated with anti-rabbit/mouse IgG conjugated to horseradish peroxidase for 2 h at room temperature, then visualized by an enhanced chemiluminescence (ECL) assay kit (Boster Biological Technology, Wuhan, China).

### Gut microbiota analysis

The microbial composition among studied groups was compared using linear discriminant analysis effect size (LEfSe) analysis. Feces were collected and washed with sterile PBS, then immediately frozen at −80°C. Fecal bacterial DNA was extracted using an E.Z.N.A.® Soil DNA Kit (Omega Bio-Tek, GA, USA). PCR amplification of 16S rRNA genes was carried out using the paired primers for the V3–V4 region of the 16S rRNA gene (supplementary Table S4, see online supplementary material). The total numbers of bacteria per gram of feces were determined by the DNA copies level of the V3–V4 region of the bacteria 16S rRNA gene. The demultiplexed reads were clustered into operational taxonomic units of >97% similarity using UPARSE. The Shannon, Simpson, and Chao indexes were used for the alpha-diversity analysis by using mothur (version 1.33.3, http://www.mothur.org/). Principal coordinate analysis (PCoA), heatmap analysis, and species abundance analysis were performed using R software (version 3.2.3, R Development Core Team, 2016). The sequencing service was provided by Bioyi Biotechnology Co., Ltd. Wuhan, China.

### RNA transcriptome analysis

Total RNA was extracted from colonic tissue by Trizol. Preparation of the cDNA transcriptome library followed the instructions of the TruSeqTM RNA sample preparation kit from Illumina (CA, USA). After qualification and quantification of cDNA, RNA sequencing was performed on a NovaSeq 6000 system from illumine. Data analysis was performed on the free online Majorbio Cloud Platform (www.majorbio.com). The raw data were subject to quality control, pre-processing, read mapping, and quantification of gene expression level. Gene Ontology (GO) enrichment and Kyoto Encyclopedia of Genes and Genomes (KEGG) pathway analysis were performed to identify the functional processes and metabolic pathways enriched by the differentially expressed genes.

### Statistical analysis

All data are expressed as the mean ± SEM. Data between two groups was compared using the unpaired t-test, and comparisons between multiple groups was carried out using ANOVA via GraphPad Prism software (version 7.01, GraphPad Prism Inc., USA). A two-sided *P* value < 0.05 was considered statistically significant.

## Results

### MyD88^−/−^ mice exhibited comparable severity of colitis to WT littermates in acute DSS-colitis

**Figure 1.**
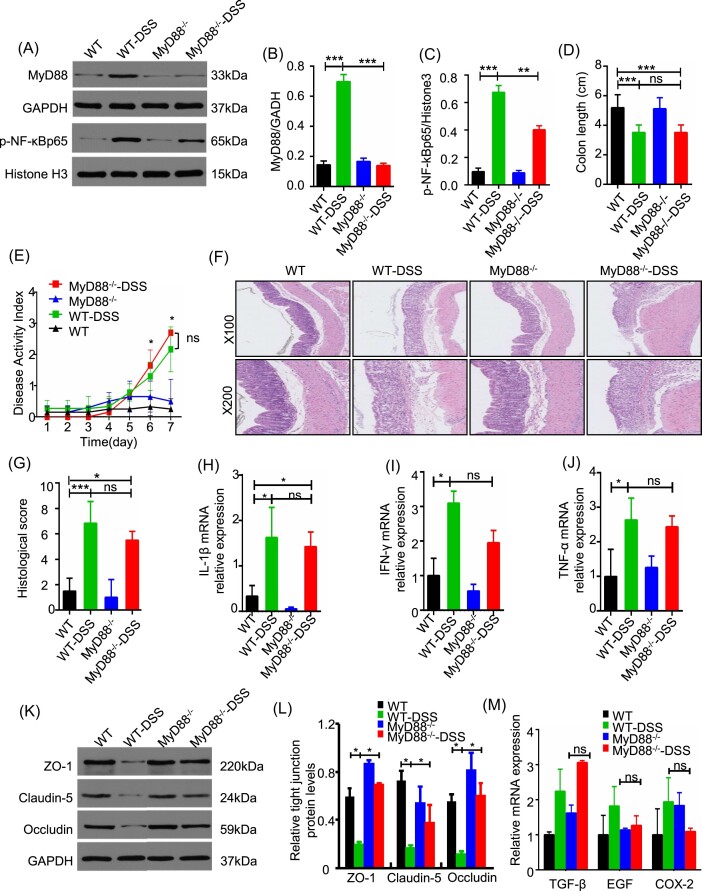
MyD88 deficiency restricted NF-κB activation but failed to alleviate colitis severity in mice. (**A**–**C**) Western blotting analysis of MyD88 protein expression and phosphorylation levels of NF-κB in colon tissue. (**D**) colon length, (**E**) DAI change, (**F**) representative H&E staining of colon sections, and (**G**) histological score of MyD88^−/−^ mice and control administrated with water or 3% DSS for 7 days. (**H**–**J**) Relative mRNA expression levels of IL-1β, IFN-γ, and TNF-α in colon tissue by RT-PCR. (**K, L**) Western blot analysis of ZO-1, claudin-5, and occludin expression in colon tissue obtained in the four groups. (**M**) Relative mRNA expression levels of TGF-β, EGF, and COX-2 in the four groups. *P < 0.05, **P < 0.01, ***P < 0.001, ns: not significant.

To investigate the involvement of MyD88 in the pathogenesis of acute colitis, we utilized MyD88-deficient mice in a DSS-induced colitis model. The administration of DSS resulted in an increase in the expression of MyD88 and activation of NF-κB in WT-DSS mice (Fig. 1A–C). Additionally, DSS exacerbated the indicators that reflect the severity of colitis, such as DAI, colonic shortening, and histological pathology (Fig. 1D–G). Compared to the WT littermates, MyD88^−/−^ mice showed less activation of NF-κB during DSS-induced colitis (Fig. 1A–C). However, there was no significant difference in DAI, colon length, and histological score between WT-DSS and MyD88^−/−^-DSS groups (Fig. 1D–G). Analysis of the mRNA expression level of TNF-α, IFN-γ, and IL-1β suggested that MyD88 deficiency could not reduce the levels of pro-inflammatory cytokines in colon caused by DSS administration (Fig. 1H–J). No considerable difference of colitis parameters was observed between MyD88^−/−^ mice and WTcontrols (Fig. 1A–J). The expression level of epithelial tight junction proteins (TJs) zona occludens-1 (ZO-1), occludin, and claudin-5 decreased in DSS-treated mice compared to the normal controls. Loss of MyD88 could partially reverse the expression of TJs after DSS administration (Fig. 1K–L). Since MyD88 was associated with tissue repair after epithelial damage, we detected the factors transforming growth factor beta (TGF-β), epidermal growth factor (EGF), and cyclooxygenase-2 (COX-2) that were involved in tissue repair within the intestinal mucosa. The results revealed that after DSS challenge, MyD88^−/−^ mice exhibited no significant differences in the expression levels of TGF-β, EGF, and COX-2 compared to the normal controls (Fig. 1M). These data indicated that MyD88 deficiency does not significantly ameliorate acute DSS-induced colitis in mice. Furthermore, MyD88-associated tissue repair does not appear to contribute to this effect.

### MyD88 inhibition failed to ameliorate the disease severity of acute DSS-induced colitis in spite of the restricted activation of NF-κB

**Figure 2.**
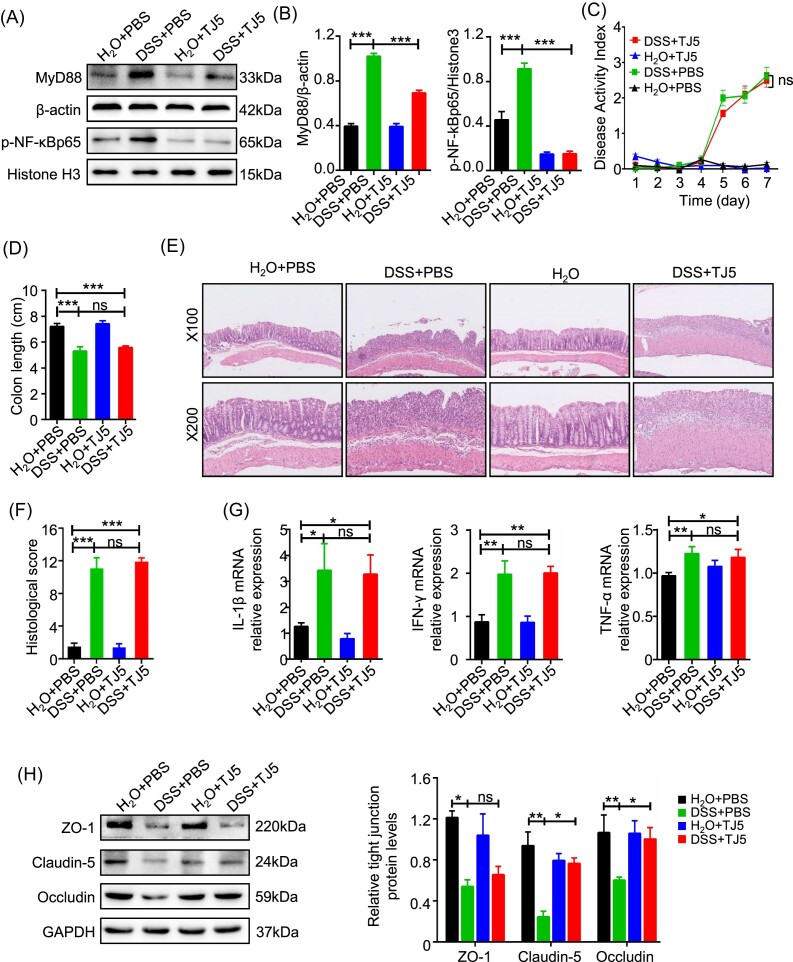
MyD88 inhibition restricted NF-κB activation but failed to alleviate acute colitis severity in mice. (**A, B**) Western blotting analysis of MyD88 protein expression and phosphorylation levels of NF-κB in colon tissue. (**C**) DAI change, (**D**) colon length, (**E**) representative H&E staining of colon sections, and (**F**) histological score of control and TJ5-treated mice administrated with water or 3% DSS for 7 days. (**G**) Relative mRNA expression levels of IL-1β, IFN-γ, and TNF-α in colon tissue by RT-PCR. (**H**) Western blot analysis of ZO-1, claudin-5, and occludin expression in colon tissue obtained from control and TJ5-treated mice administrated with water or 3% DSS for 7 days. Data are presented as mean ± SEM; *P < 0.05, **P < 0.01, ***P < 0.001, ns: not significant.

We then tested the effects of the MyD88 inhibitor (TJ5) on acute intestinal inflammation. Western blot analysis showed that TJ5 administration could significantly restrict the expression of MyD88 in colon tissue. In the colons of mice with DSS-induced colitis, TJ5 treatment resulted in a significantly decreased level of phosphorylated NF-κB compared to mice treated with PBS (Fig. 2A and B).

Mice challenged with DSS for 7 days had increased DAI, worse histological score, and shortened colon compared with mice without DSS challenge no matter whether TJ5 was administrated or not (Fig. 2C–F). Moreover, the expression of proinflammatory cytokines including TNF-α, IFN-γ, and IL-1β was not significantly suppressed in TJ5-treated mice (Fig. 2G). Suppression of MyD88 by TJ5 could partially reverse the expression of TJs ZO-1, occludin, and claudin-5 after DSS administration (Fig. 2H), but no considerable differences were observed in the expression levels of TGF-β, EGF, and COX-2 compared to the normal controls after DSS challenge (supplementary Fig. S1, see online supplementary material).

Taken together, these data suggested that TJ5 could inhibit My88 expression and partially suppress the activation of NF-κB. However, inhibition of MyD88 did not significantly ameliorate the disease severity of acute DSS-induced colitis, which was in concordance with data from MyD88-deficient mice.

### MyD88 inhibition disturbed the composition of intestinal microflora in mice

**Figure 3.**
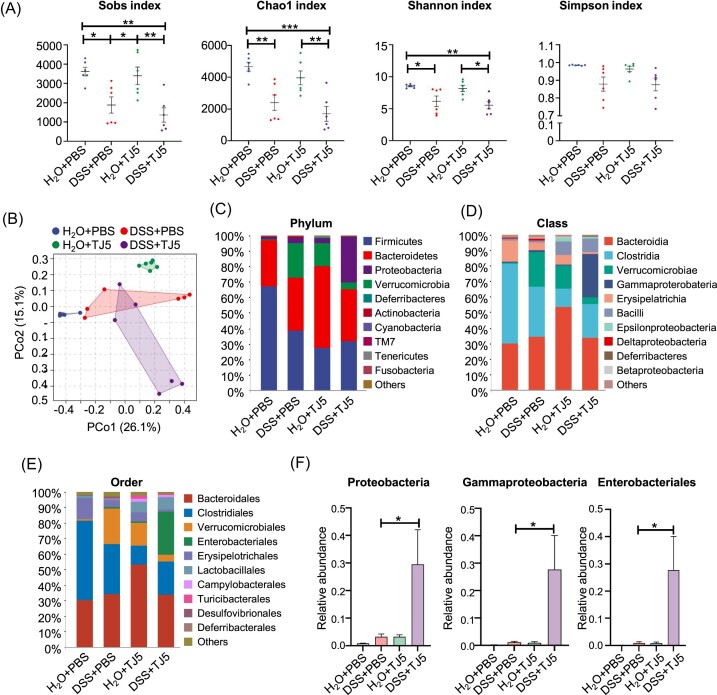
Effects of MyD88 inhibition on the gut microbiota of mice. (**A**) Rarefaction plots based on the Sobs, Chao1, Shannon, and Simpson indexes. (**B**) PCoA plot of the intestinal mucosa-associated microbiota with Bray–Curtis distance. Average relative abundances of taxa at the (**C**) phylum level, (**D**) class level, and (**E**) order level. (**F**) Relative abundances of indicated bacterial taxa in feces. Data are presented as mean ± SEM, *P < 0.05, **P < 0.01, ***P < 0.001.

We next analyzed the possible reasons for the comparable colitis severity between MyD88-suppressed mice and their control animals after DSS challenge. In light of the important role of MyD88 in the interaction between gut microbiota and host immunity, we examined the effect of MyD88 suppression on the gut microbial ecosystem. The analysis of 16S rDNA sequencing in fecal samples unveiled significant variations in bacterial diversity and abundance indexes (Sobs, Chao1, Shannon, and Simpson) across the H_2_O + PBS, DSS + PBS, H_2_O + TJ5, and DSS + TJ5 experimental groups. (Fig. 3A). PCoA analysis revealed that the composition of gut microbiota separated significantly in the four groups (Fig. 3B). At the phylum level, MyD88-suppressed mice with TJ5 treatment showed an increase in *Bacteroidetes* and *Proteobacteria*, and a decrease in *Firmicutes* in the colon compared to the PBS-treated control. A similar trend of increased *Proteobacteria* and decreased *Firmicutes* was also observed in MyD88-deficient mice (supplementary Fig. S2, see online supplementary material). These data revealed that MyD88 suppression led to an unfavorable alteration in the composition of gut microbiota. In DSS-induced colitis, the abundance of *Proteobacteria* was increased in TJ5-treated mice as compared to PBS-treated mice (Fig. 3C and F, and supplementary Fig. S3, see online supplementary material). At the class level, the dominant bacterial community of PBS-treated mice was *Clostridia*. Compared with the PBS-treated mice, TJ5-treated mice showed reduced *Clostridia* and increased *Bacteroidia* (Fig. 3D). Compared to the DSS + PBS group, the abundance of *Gammaproteobacteria* significantly increased in the DSS + TJ5 group (Fig. 3D and F). Within the *Proteobacteria* phylum, *Enterobacteriales* were predominantly increased at order level in TJ5-treated mice (Fig. 3E and F), whereas *Clostridiales*, which belong to *Firmicutes*, exhibited a significant decrease in TJ5-treated mice (Fig. 3E). These data revealed that MyD88 suppression leads to an unfavorable alteration in the composition of gut microbiota.

### NLR signaling pathway was the major immune-related pathway upregulated in DSS-induced colitis mice treated with TJ5

**Figure 4.**
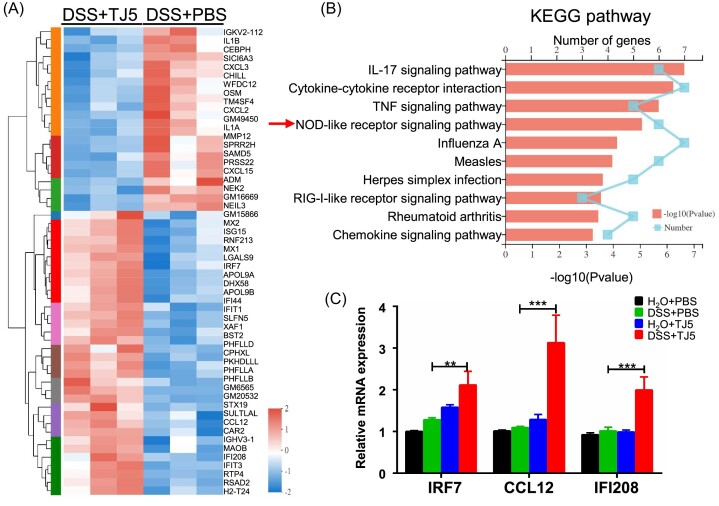
MyD88 inhibition induced the upregulation of the NOD-like receptor signaling pathway in mice with DSS-induced colitis. (**A**) RNA-sequencing was performed with isolated colon tissue from mice with DSS-induced colitis treated with PBS or TJ5. The heat map indicated the differentially expressed genes by clustering analysis. (**B**) Differentially expressed genes (DSS + PBS group versus DSS + TJ5 group) were analyzed by KEGG enrichment analysis. (**C**) Differentially expressed genes (DSS + PBS group versus DSS + TJ5 group) associated with the NLR signaling pathway were identified by RT-PCR. Data are presented as mean ± SEM, **P < 0.01, ***P < 0.001.

To explore the signaling pathways possibly mediating the unmitigated colitis in DSS-fed mice after MyD88 suppression, we performed RNA transcriptome analysis of colon tissue from the DSS + TJ5 mice versus the DSS + PBS mice. The comparative analysis revealed that 56 genes were differentially expressed between the two groups (Fig. 4A). The differentially expressed genes were subjected to additional GO analysis and KEGG enrichment analysis. GO analysis showed that MyD88 suppression in acute DSS-induced colitis was related to the defense response, immune response and response to organism/external biotic stimulus (supplementary Fig. S4, see online supplementary material). KEGG pathway analysis showed that immune-related pathways, including the IL-17, TNF, and NLR signaling pathways were the major pathways associated with MyD88 inhibition in DSS-induced colitis (Fig. 4B). Real-time PCR confirmed that only genes related to the NLR signaling pathway exhibited enhanced mRNA expression in DSS + TJ5 mice compared to the DSS + PBS mice (Fig. 4C and supplementary Figs. S5–S7, see online supplementary material).

### Further suppression of NLR signaling pathway ameliorated the unmitigated colitis severity in TJ5 treated DSS-colitis mice

**Figure 5.**
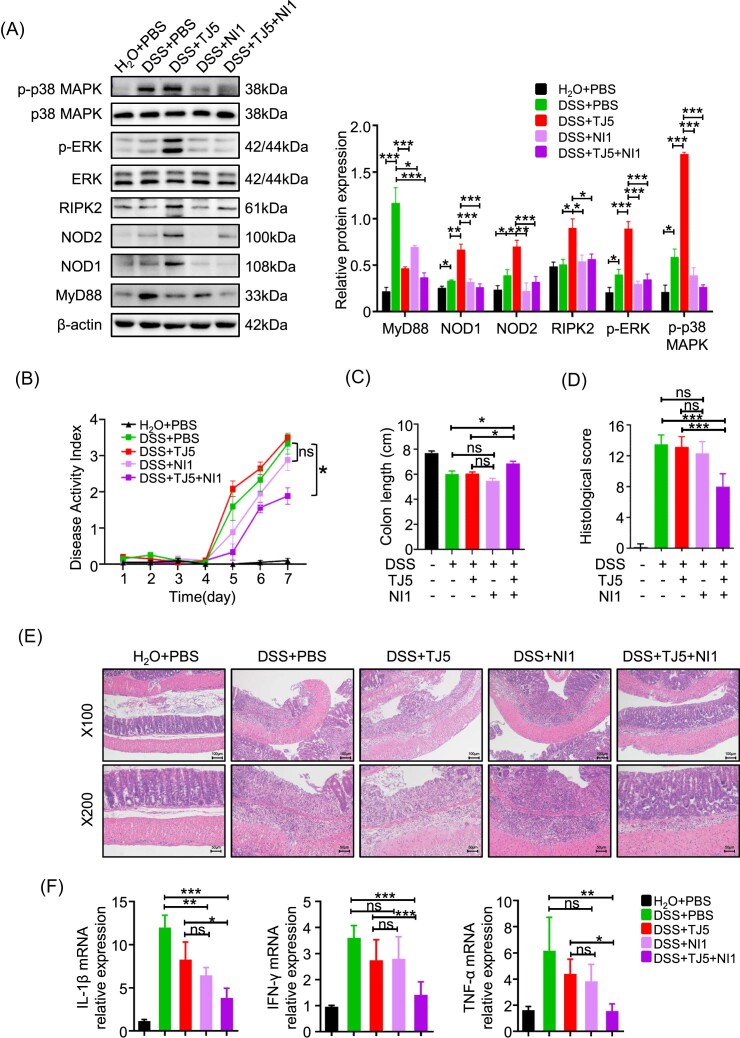
Further blockade of the NLR signaling pathway attenuated unmitigated DSS-colitis severity in mice treated with TJ5 alone. (**A**) Colon samples were collected after 7 days of DSS administration. Protein extracted from colon tissue was analyzed by western blotting for MyD88, NOD1/2, extracellular regulated protein kinases (ERK), phospho-ERK, p38 MAPK, and phospho-p38 MAPK. (**B**) DAI change, (**C**) colon length, and (**D**) histological score of mice in different groups. (**E**) Representative H&E staining of colon sections. (**F**) Relative mRNA expression levels of IL-1β, IFN-γ, and TNF-α in colon tissue by RT-PCR. Data are presented as mean ± SEM, *P < 0.05. **P < 0.01, ***Pp < 0.001, ns: not significant.

The above results demonstrated that MyD88 inhibition could not alleviate colitis severity and was associated with the alteration of gut microbiota composition and upregulation of the NLR signaling pathway in mice with DSS challenge. To better understand the role of the NLR signaling pathway in colitis under MyD88 inhibition conditions, TJ5-treated mice were intraperitoneally injected with NLRs inhibitor NI1. Western blot analysis indicated that the protein expression levels of NLRs and NLR-induced mitogen activated protein kinase (MAPK) signal were significantly upregulated in the DSS + TJ5 group compared with the DSS + PBS group. Administration of NI1 could suppress the upregulation of the NLR signal in mice with DSS-induced colitis (Fig. 5A). Meanwhile, mice from the DSS + TJ5 + NI1 group displayed significantly decreased DAI, HS, and colon shortening than those from the DSS + PBS group or the DSS + TJ5 group (Fig. 5B–E). The mRNA expression of proinflammatory cytokines in colonic tissues, including IL-1β, IFN-γ, and TNF-α, were decreased in DSS-induced colitis mice treated with NI1 and TJ5 as compared to mice solely treated with TJ5 (Fig. 5F). The data suggested that further inhibition of the NLR signaling pathway in TJ5-treated DSS-colitis mice could ameliorate colitis severity, which was not improved by MyD88 inhibition with TJ5 alone.

### Antibiotics treatment altered colonic bacterial flora in mice

**Figure 6.**
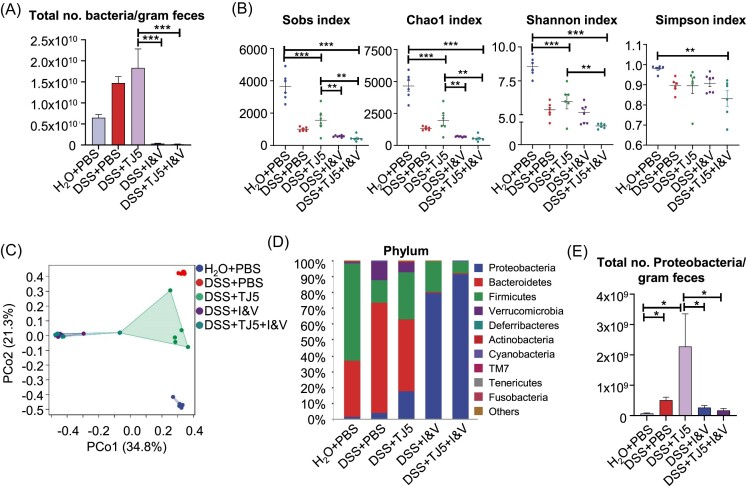
Alteration of colonic bacterial flora by broad-spectrum antibiotics in mice. Animals were given imipenem (I; 1g/l) and vancomycin (V; 1g/l) in drinking water for 3 days before and during DSS administration. (**A**) Total number of bacteria per gram of feces was quantified using RT-PCR. (**B**) Rarefaction plots based on the Sobs, Chao1, Shannon, and Simpson indexes. (**C**) PCoA plot of the intestinal mucosa-associated microbiota with Bray–Curtis distance. (**D**) Average relative abundances of taxa at the phylum level. (**E**) Total number of *Proteobacteria* per gram of feces in different groups. Data are presented as mean ± SEM, *p < 0.05. **p < 0.01, ***p < 0.001.

Considering the important role of MyD88 in the interaction between gut microbiota and host immunity, we next examined the combination effect of MyD88 suppression and antibiotics on the gut microbial ecosystem. Animals were given imipenem (I; 1 g/l) and vancomycin (V; 1 g/l) in drinking water for 3 days before and during DSS administration to eliminate the gut microflora. The total number of bacteria per gram of feces was much less after antibiotics treatment (Fig. 6A). Analysis of colonic mucosal samples via 16S rDNA sequencing revealed significant differences in the diversity and abundance indexes of microbiota, such as Sobs, Chao1, Shannon, and Simpson, among the H_2_O + PBS, DSS + PBS, DSS + TJ5, DSS + I&V, and DSS + TJ5 + I&V groups (Fig. 6B). PCoA analysis revealed that the composition of colonic microbiota differed significantly in the five groups (Fig. 6C). In DSS-induced colitis mice at the phylum level, TJ5-treated and/or I&V-treated mice showed an increase in *Proteobacteria* and a decrease in Bacteroidetes in the composition of the colonic microbiota compared to the PBS-treated mice (Fig. 6D). Among the five groups, the largest total number of *Proteobacteria* per gram of feces was observed in the TJ5-treated mice (Fig. 6E). However, the total number of *Proteobacteria* per gram of feces exhibited a significant decrease after antibiotic treatment. (Fig. 6E). These data revealed that the effect of MyD88 suppression on gut microbiota could be altered after antibiotics treatment.

### Antibiotics treatment impeded colitis progression in MyD88-suppressed DSS-colitis mice

**Figure 7.**
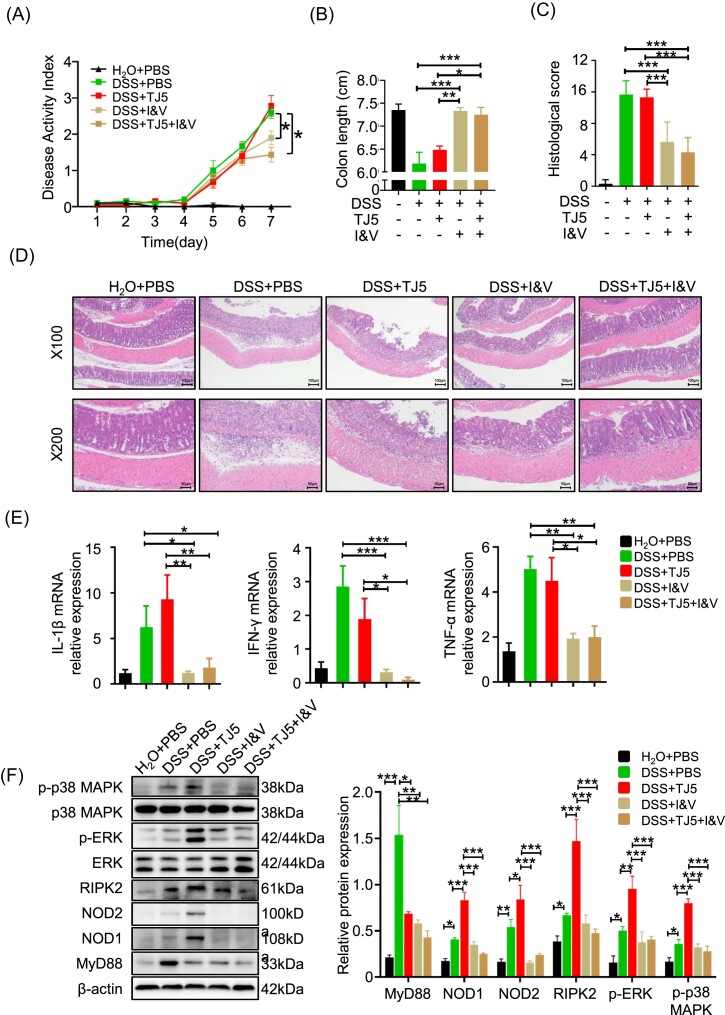
Antibiotics treatment dampened colitis progression in TJ5-treated mice. (**A**) DAI change, (**B**) colon length, and (**C**) histological score of mice. (**D**) Representative H&E staining of colon sections from mice in each group. (**E**) Relative mRNA expression levels of IL-1β, IFN-γ, and TNF-α in colon tissue by RT-PCR. (**F**) Protein extracted from colon tissue was analyzed by western blot for MyD88, NOD1/2, RIPK2, ERK, phospho-ERK, p38 MAPK, and phospho-p38 MAPK. Data are presented as mean ± SEM, *p < 0.05, **p < 0.01, ***p < 0.001, I&V: imipenem and vancomycin.

We hypothesized that the MyD88 inhibition-associated increase in *Proteobacteria* in the gut might upregulate the NLR signaling pathway, potentially leading to unmitigated colitis severity following MyD88 inhibition. In our study, TJ5-treated mice were orally given broad-spectrum antibiotics with a combination of imipenem and vancomycin to eliminate gut bacteria. Compared to PBS- or TJ5-treated DSS-colitis mice, the antibiotics plus TJ5-treated mice exhibited significantly lower DAI, histological scores, and less colon shortening after DSS administration (Fig. 7A–C). Pathological findings and proinflammatory cytokine levels revealed ameliorated colonic inflammation in TJ5-treated DSS-induced colitis mice after antibiotic treatment (Fig. 7D–E). Using broad-spectrum antibiotics to limit gut bacteria resulted in a significant decrease in the expression level of NLRs and activated MAPK proteins in TJ5-treated mice with DSS-colitis (Fig. 7F). The data suggest that the increase in gut *Proteobacteria* in MyD88-suppressed mice may exacerbate intestinal inflammation by stimulating NLR-mediated inflammatory responses.

## Discussion

**Figure 8.**
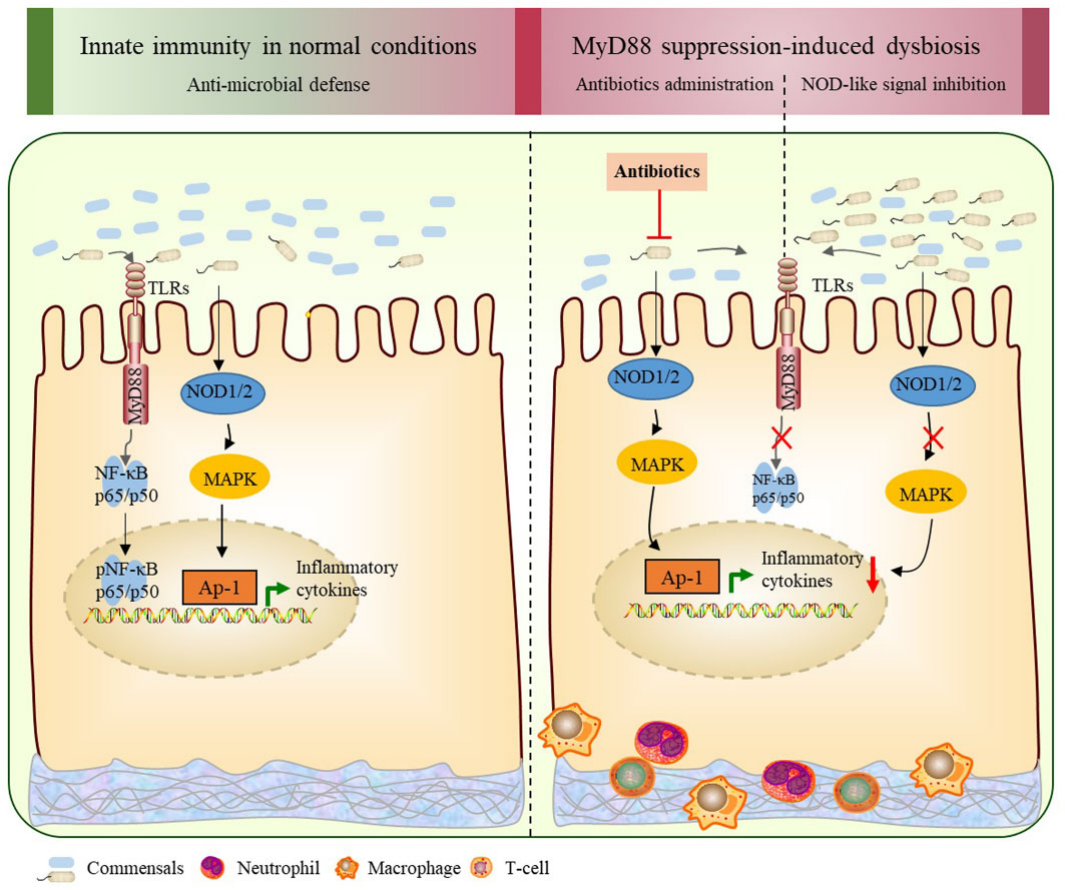
Schematic diagram depicting gut dysbiosis and stimulation of the NLR signaling pathway after MyD88 suppression in intestinal inflammation. In normal conditions, the extracellular and intracellular PPRs (TLRs and NLRs) sense intestinal microbiota through PAMPs and exert anti-bacterial effects. In the state of intestinal inflammation, although MyD88 suppression can restrict the MyD88-mediated activation of NF-κB, it also leads to unfavorable changes in the composition of gut microbiota and induces the upregulation of the NLR signaling pathway. Eliminating gut microbiota with antibiotics or blocking NLR signal transduction with NLR inhibitor in combination with MyD88 suppression can ameliorate the inflammatory response.

Suppression of the overactive immune response has been regarded as an important therapeutic strategy for IBD. Our study suggests that inhibiting MyD88 with TJ5 may not improve the severity of colitis in an acute DSS-induced colitis model, even though the activation of the proinflammatory NF-κB pathway was partially restricted. The results imply that the unfavorable changes in the gut microbiota and the upregulation of the NLR signaling pathway after MyD88 suppression could account for the sustained DSS-associated colonic inflammation and intestinal mucosal injury following MyD88 inhibition (Fig. 8).

The MyD88-mediated innate immune response was suggested to be an important promoter of intestinal inflammation and could potentially serve as a therapeutic target for IBD. In a mouse model of azoxymethane/DSS-induced colitis-associated cancer, administration of a MyD88 inhibitor significantly alleviated azoxymethane/DSS-inaduced colitis, resulting in reduced body weight loss and lower mortality rates [24]. Furthermore, in the IL10^−/−^ mouse model of chronic intestinal inflammation, the deletion of MyD88 protected IL10^−/−^ mice from spontaneous commensal-dependent colitis [25]. Interestingly, our study demonstrated that blocking the MyD88-mediated immunological pathway alone could not ameliorate DSS-induced colitis severity in terms of body weight loss, DAI, histological change, and several proinflammatory cytokine levels including TNF-α, IFN-γ, and IL-1β, although the activation of NF-κB was significantly inhibited. This result was similar to the observations in several other recent studies. Araki [19] and Rakoff-Nahoum [20] found that MyD88-deficient mice exhibited increased susceptibility to DSS, resulting in severe mucosal injury, rectal bleeding, and even higher mortality. Mice with intestinal epithelial-specific blockade of MyD88 even developed spontaneous inflammation in the small intestine at 36 weeks [26]. These contradictory results of MyD88 blockade in the above murine models of intestinal inflammation may be attributed to the complex role of MyD88 in intestinal inflammation.

The adaptor MyD88 could mediate the activation of the NF-κB signaling pathway and was classically defined as a booster of mucosal inflammatory responses [11]. Besides, MyD88 was important in regulating epithelial repair and mucosal permeability [18, 20]. Our results indicated that MyD88 suppression could restrict the activation of NF-κB but could not reduce the levels of several proinflammatory cytokines, including IL-1β, IFN-γ, and TNF-α. mRNA expression levels of tissue repair factors, including TGF-β, EGF, and COX-2 within the intestinal mucosa, were not significantly decreased after MyD88 suppression. Additionally, the integrity of TJs could be partially preserved by MyD88 inhibition following DSS administration. These results suggest that the suppression of MyD88 in experimental colitis could restrict the activation of NF-κB without compromising tissue reconstruction ability and the production of epithelial TJs. Further investigation was conducted to explore the possible explanation as to why inhibiting MyD88 did not alleviate the severity of acute DSS-induced colitis.

MyD88 plays a key role in the innate immune defense against pathogens, and it has been reported that autosomal recessive MyD88 deficiency in children is associated with increased susceptibility to pyogenic bacterial infection [27]. Production of anti-bacterial agents such as antimicrobial peptides and secretory immunoglobulin A (slgA) also required MyD88 [26]. As the key adaptor of TLRs, MyD88 may participate in TLR-mediated shaping and regulating of gut microbiota [28, 29]. Given that MyD88 is involved in multiple antibacterial defense pathways, the hypo-expression of MyD88 may lead to the accumulation of pathogens in the gut and disrupt the structure of the gut microbiota. Our findings revealed that MyD88-suppressed mice had unfavorable alterations in gut microbial composition, characterized by an increased abundance of *Proteobacteria. Proteobacteria* is a minor phylum in the human gut, consisting of several known human pathogens, such as *Escherichia, Shigella, Salmonella*, and *Yersinia*. These pathogens can activate the host immune defense and trigger a proinflammatory state [30, 31]. Patients with IBD were often found to have increased *Proteobacteria* in the gut [32 – 34]. Thus, MyD88 suppression may lead to dysbiosis and stimulate intestinal inflammation by activating other immunological pathways.

In our study, RNA transcriptome analysis showed that the NLR signaling pathway was the primary upregulated immunological pathway associated with MyD88 suppression in DSS-induced colitis. Further blockade of the NLR signaling pathway in MyD88-suppressed mice could ameliorate the severity of colitis that was not improved by MyD88 inhibition. Besides, the elimination of intestinal *Proteobacteria* in MyD88-suppressed mice by broad-spectrum antibiotics could also alleviate colitis severity. This effect is coupled with the reduced expression of NLRs and the inhibition of the NLR-mediated MAPK signaling pathway. It was acknowledged that both NLRs and TLRs were important families of PRRs, which collaborated to recognize PAMPs and induce immune defense against invading pathogens in intestinal epithelia [35]. The NLRs are localized in the cytoplasm and have been shown to play a key role in defense against intracellular microbiota. PAMPs derived from pathogens can activate NLRs, leading to downstream proinflammatory signal transduction and cytokine production [9, 36]. This implies that the inhibition of MyD88 and the dysbiosis related to hypo-expression of MyD88 can induce the activation of the NLR signaling pathway in acute DSS-induced colitis. This could be the reason why the inhibition of MyD88 failed to mitigate the severity of colitis, as evaluated by DAI, HS, and several proinflammatory cytokines, including IL-1β, IFN-γ, and TNF-α.

## Conclusion

In conclusion, this study indicates that the blockade of the MyD88-mediated immunological pathway is associated with unfavorable changes in gut microbiota, leading to the induction of NLR-mediated immune responses. The cooperation of PRRs is crucial for modulating intestinal inflammation.

## Supplementary Material

pbae013_Supplemental_File

## Data Availability

The datasets supporting the conclusions of this article are included within the article and the sequencing data in this study are available in the NCBI online repository (https://www.ncbi.nlm.nih.gov/, accession numbers: PRJNA904645 and PRJNA904649).
