# Supplementary material for: Suppression of MyD88 disturbs gut microbiota and activates the NLR pathway and hence fails to ameliorate DSS-induced colitis
Source: Precis Clin Med. 2024 Jun 6;7(2):pbae013. doi: 10.1093/pcmedi/pbae013 (PMC11212664; doi:10.1093/pcmedi/pbae013)
Supplement: pbae013_Supplemental_File [file pbae013_supplemental_file.pdf]

Supplementary Table 1. Scoring criteria for disease activity index (DAI)*

Score	Weight loss (%)	Stool consistency	Occult/gross bleeding
0	(-)	Normal	Normal
1	1-5	Loose	
2	6-10	Loose	Guica(+)
3	11-15	Diarrhea	
4	>15	Diarrhea	Gross bleeding

*DAI=score (weight loss + stool consistency + bleeding) /3
*Normal stools well formed pellets; loose pasty stools which do not stick to the anus; diarrhea liquid stools that stick to the anus.

Supplementary Table 2. Histological grading of colitis

Feature graded	Grade	Description
Inflammatory cell infiltration	0	Occasional inflammatory cells in the lamina propria
	1	Increased infiltrate in the lamina propria predominantly at the base of crypts
	2	Confluence of inflammatory infiltrate extending into the mucosa
	3	Transmural extension of infiltrate
Inflammation extent	0	None
	1	Mucosa
	2	Mucosa and submucosa
	3	Transmural
Crypt damage	0	None
	1	Basal 1/3 damaged
	2	Basal 2/3 damaged
	3	Only surface epithelium intact
	4	Entire crypt and epithelium lost
Percent involvement	1	1-25%
	2	26-50%
	3	51-75%
	4	76-100%

Supplementary Table 3. Primers used for RT-PCR

Primier	Sequence
IL-1β	Forward: 5’-ACTCCTTAGTCCTCGGCCA-3’
	Reverse: 5’-TGGTTTCTTGTGACCCTGAGC-3’
TNF-α	Forward: 5’-CAGGCGGTGCCTATGTCTC-3’
	Reverse: 5’-CGATCACCCCGAAGTTCAGTAG-3’
IFN-γ	Forward: 5’-GCCACGGCACAGTCATTGA-3’
	Reverse: 5’-TGCTGATGGCCTGATTGTCTT-3’
MyD88	Forward: 5’-TCATGTTCTCCATACCCTTGGT-3’
	Reverse: 5’-AAACTGCGAGTGGGGTCAG-3’
ZO-1	Forward: 5’-GCCGCTAAGAGCACAGCAA-3’
	Reverse: 5’-GCCCTCCTTTTAACACATCAGA-3’
Claudin-5	Forward: 5’-GCAAGGTGTATGAATCTGTGCT-3’
	Reverse: 5’-GTCAAGGTAACAAAGAGTGCCA-3’
Occludin	Forward: 5’-TGAAAGTCCACCTCCTTACAGA-3’
	Reverse: 5’-CCGGATAAAAAGAGTACGCTGG-3’
TGF-β	Forward: 5’-CCACCTGCAAGACCATCGAC-3’
	Reverse: 5’-CTGGCGAGCCTTAGTTTGGAC-3’
EGF	Forward: 5’-TTAACGGGACAGGACTAGAGAAA-3’
	Reverse: 5’-AAGGAACTTAGAAGAACTCGGGA-3’
COX-2	Forward: 5’-CCACCTCTGCGATGCTCTTC-3’
	Reverse: 5’-ACATTCCCCACGGTTTTGAC-3’
IL-17	Forward: 5’-TTTAACTCCCTTGGCGCAAAA-3’
	Reverse: 5’-CTTTCCCTCCGCATTGACAC-3’
IRF7	Forward: 5’-GCGTACCCTGGAAGCATTTC-3’
	Reverse: 5’-GCACAGCGGAAGTTGGTCT-3’
CCL2	Forward: 5’-TAAAAACCTGGATCGGAACCAAA-3’
	Reverse: 5’-GCATTAGCTTCAGATTTACGGGT-3’
IFI16	Forward: 5’-AAAGGAGCCTGCTAAGGAAGA-3’
	Reverse: 5’-CGTTCACATCAGAGACACAGGA-3’
GAPDH	Forward: 5’-AGGTCGGTGTGAACGGATTTG-3’
	Reverse: 5’-GGGGTCGTTGATGGCAACA-3’

Supplementary Table 4. Primers for the V3-V4 region of the 16S rRNA gene

**Primier**	**Sequence**
338F	Forward: 5’-ACTCCTACGGGAGGCAGCAG-3’
806R	Reverse: 5’-GGACTACHVGGGTWTCTAAT-3’

Supplementary Figure 1

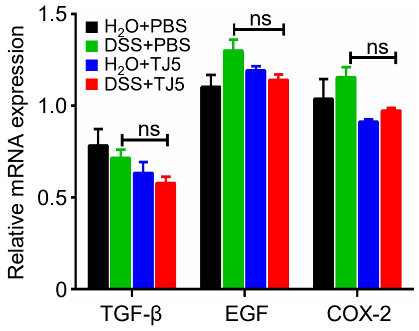

Supplementary Figure 1. MyD88 inhibition failed to interfere with the intestinal epithelial reconstruction ability. Relative mRNA expression levels of TGF-β1, EGF, and COX-2 in colon tissue of control and TJ5-treated mice by RT-PCR analysis. Data were represented as mean ± SEM, *p<0.05, ns: not significant. TJ5: TJ-M2010-5.

Supplementary Figure 2

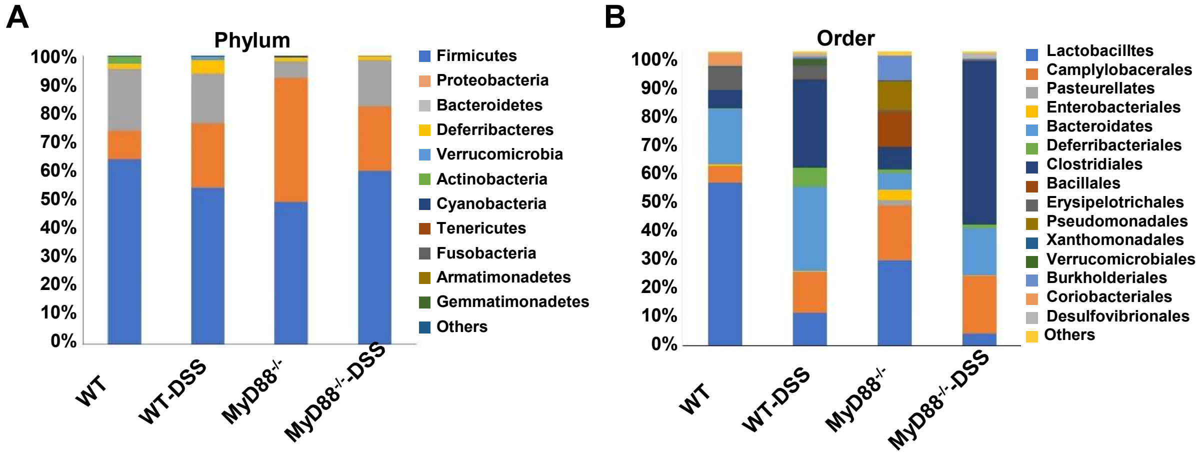

Supplementary Figure 2. Effects of MyD88 deficiency on the intestinal microflora in mice. Average relative abundances of taxa at the phylum level (A) and order level (B) among four groups.

Supplementary Figure 3

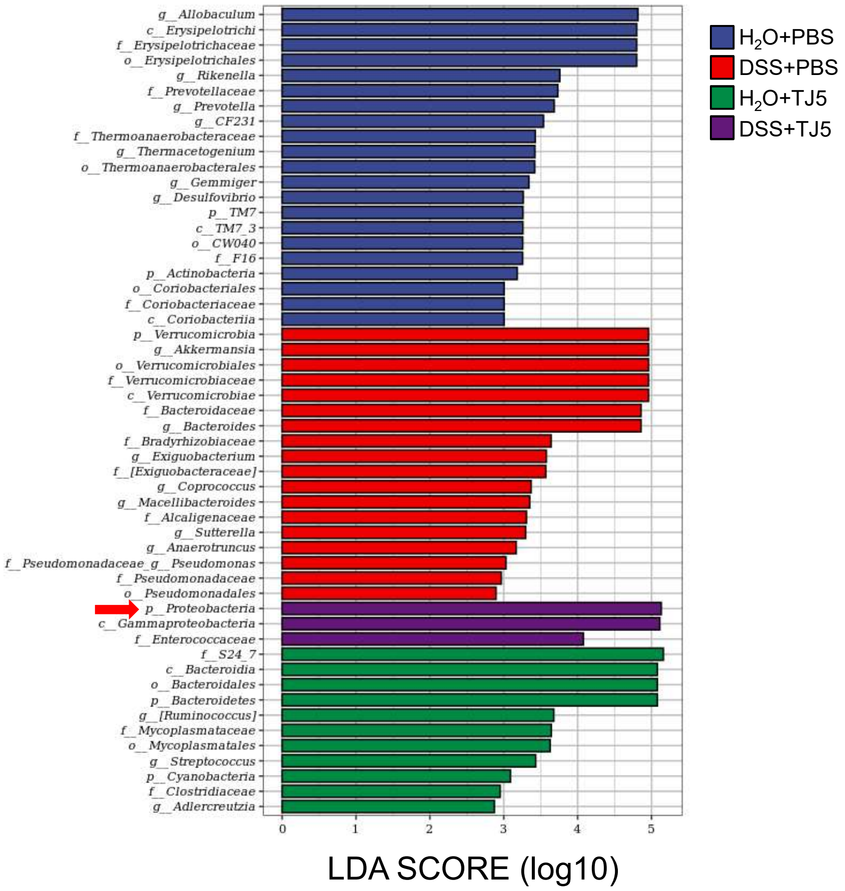

Supplementary Figure 3. Significant changes in functional profiles were suggested based on phylogenetic investigations of communities through LEfSe analyses (LDA-score >2.5). The dominating taxa in DSS+TJ5 group were enriched in *Proteobacteria* at the phylum level.

Supplementary Figure 4

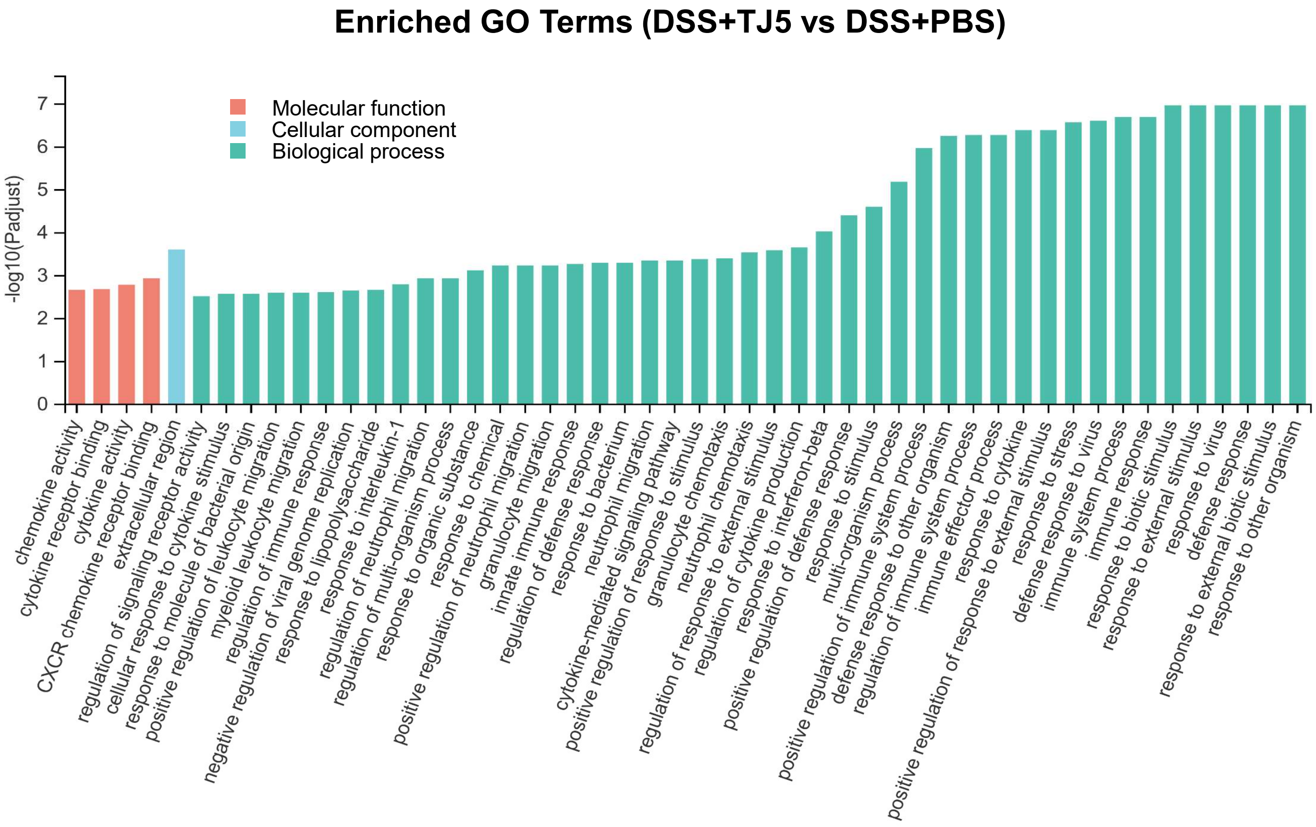

Supplementary Figure 4. The differentially expressed genes of DSS+TJ5 group compare with DSS+PBS group were analyzed by GO.

Supplementary Figure 5

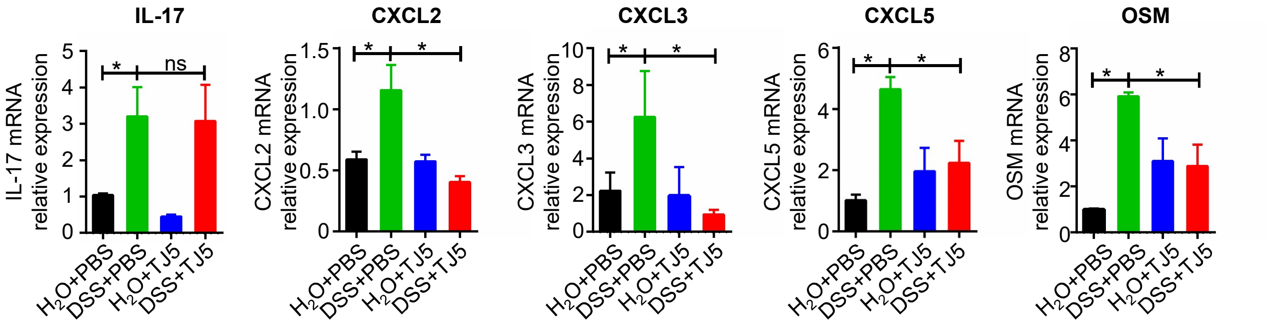

Supplementary Figure 5. The differentially expressed genes (DSS+PBS group vs. DSS+TJ5 group) associated with NOD-like receptor signaling pathway were identified by RT-PCR. Data were represented as mean ± SEM, *p<0.05, ns: not significant. TJ5: TJ-M2010-5.

Supplementary Figure 6

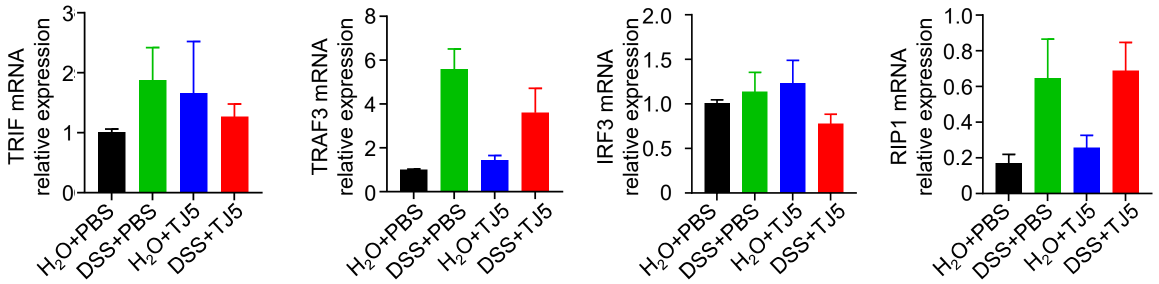

Supplementary Figure 6. No significant difference was detected in the key genes involved in the MyD88-independent signaling pathway by RT-PCR. TJ5: TJ-M2010-5.

Supplementary Figure 7

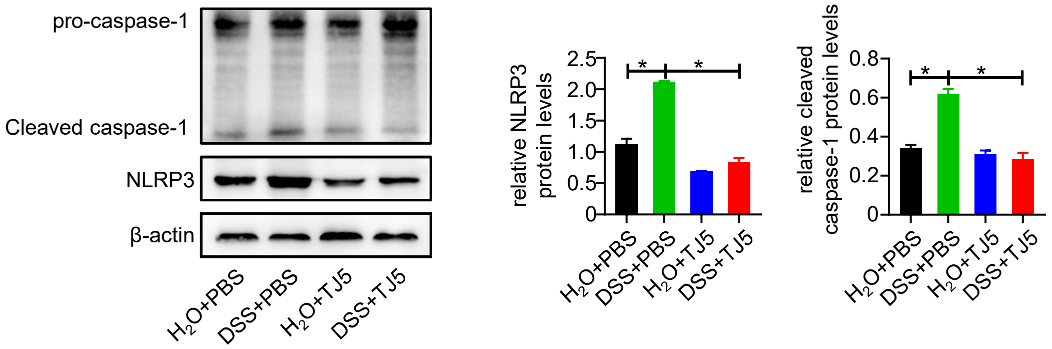

Supplementary Figure 7. NLRP3 signaling activation was suppressed by TJ5 administration determined by western blot analysis. Data were represented as mean ± SEM, *p<0.05, ns: not significant. TJ5: TJ-M2010-5.
